# Contextualizing Evidence for Action on Diabetes in Low-Resource Settings—Project CEAD Part-II, Strengthening the Health System: A Mixed-Methods Study Protocol

**DOI:** 10.3390/ijerph18073391

**Published:** 2021-03-25

**Authors:** Mari Carmen Bernal-Soriano, Francisco Barrera-Guarderas, Alfonso Alonso-Jaquete, Elisa Chilet-Rosell, Ikram Benazizi, Cintia Caicedo-Montaño, Mónica Márquez-Figueroa, Marta Puig-García, Blanca Lumbreras, Ildefonso Hernández-Aguado, Ana Lucía Torres-Castillo, Lucy Anne Parker

**Affiliations:** 1 Department of Public Health, Universidad Miguel Hernández, Sant Joan d’Alacant, 03550 Alicante, Spain; echilet@umh.es (E.C.-R.); ibenazizi@umh.es (I.B.); marta.puig@alu.umh.es (M.P.-G.); blumbreras@umh.es (B.L.); ihernandez@umh.es (I.H.-A.); lparker@umh.es (L.A.P.); 2 CIBER de Epidemiología y Salud Pública (CIBERESP), 28029 Madrid, Spain; 3 Faculty of Medicine, Pontificia Universidad Católica del Ecuador (PUCE), Quito 170143, Ecuador; jfbarrera@puce.edu.ec; 4 Unidad Docente de Medicina Preventiva y Salud Pública de Cantabria, Consejería de Sanidad de Cantabria, 39009 Santander, Spain; aalonjaq@gmail.com; 5 Centre of Community Epidemiology and Tropical Medicine (CECOMET), Esmeraldas 0801265, Ecuador; cintiacaicedo@cecomet.org (C.C.-M.); monicamarquez@cecomet.org (M.M.-F.); 6 Institute of Public Health, Pontificia Universidad Católica del Ecuador (PUCE), Quito 170143, Ecuador; atorres331@puce.edu.ec

**Keywords:** implementation science, diabetes mellitus, diabetes type 2, public health, health systems, low- and middle-income countries

## Abstract

Diabetes is a major public health problem, increasingly affecting low- and middle-income countries. The project CEAD (Contextualizing Evidence for Action in Diabetes in low-resource settings) aims to evaluate the implementation of comprehensive diabetes care in two low-resource settings in Ecuador and to stimulate context-led health systems innovations to improve diabetes care and reduce inequity. The mixed-methods approach includes a 24-month retrospective study to assess the current level of implementation of comprehensive diabetes care and participants will be followed up prospectively for two years to assess changes in healthcare and clinical outcomes from the outset of the research. We will include individuals diagnosed with type-2 diabetes aged over 18 years, who are accessing diabetes care in health facilities in the study districts. Varied stakeholders (patients and family members, community members, healthcare workers and decision-makers) will interpret the underlying causes of the observed weaknesses and propose solutions to strengthen diabetes-related healthcare in focus group discussions (FG). A second set of FG will analyze perceived improvements in healthcare based on prospective cohort findings and consider the success/failure of any context-led innovations occurring throughout the research. Our study will demonstrate how evidence can be contextualized to stimulate local innovations and overcome weaknesses of diabetes-related healthcare in low resource settings.

## 1. Introduction

Noncommunicable diseases (NCDs), such as diabetes, are a major global public health problem and are closely linked to poverty [1]. On one hand, NCDs are more prevalent among low-income population groups with NCDs, and on the other hand, people with NCDs have additional challenges in productivity which can impact economic development. Diabetes can lead to major health complications, as well as increasing the risk of all-cause mortality, especially among younger people of working age [2]. The growing burden of NCDs in low- and middle-income countries (LMICs) is threatening the capacity of health systems to respond adequately to the population’s needs [3]. Not only do LMICs bear 86% of the burden of NCD-related premature deaths [4], but they are home to 75% of people living with diabetes [5].

Diabetes treatment includes blood glucose control through a combination of diet and physical activity and, if necessary, medication. In addition, it is necessary to control blood pressure and lipids to reduce cardiovascular risk and carry out regular screening for retinopathy, nephropathy and diabetic foot to facilitate early treatment of diabetes complications [2]. Given that most evidence on how to manage and prevent NCDs is largely generated in high-income settings [6], it is possible that elements of successful control, such as the availability of a multidisciplinary team trained in diabetes management, or periodic assessment by medical specialists, may be difficult to apply in low-resource settings.

In this regard, a recently published study [7] found a total unmet need for diabetes care of 77% in low- and middle-income countries. Deficits were identified at diagnosis with a lack of testing, and during monitoring with sub-optimal levels of glycemic control. Most countries have national diabetes policies, but their level of implementation is variable. Less than half of countries with national guidelines for diabetes management report their full implementation [8]. For example, several studies show shortcomings in the implementation of key processes of diabetes care and glycemic control in LMICs [9,10]. Therefore, there is a need to develop clinical guidelines for chronic diseases that can be realistically applied in resource-constrained primary care settings [9]. In Ecuador, where diabetes was the second cause of death in 2018 [11], the Ministry of Public Health published its first Clinical Practice Guideline (CPG) for Type 2 Diabetes in 2017 [12]. This guideline includes recommendations for multidisciplinary disease management at primary, secondary and tertiary levels. It is an adaptation of the United Kingdom’s National Institute of Clinical Excellence (NICE) guidance [13]. However, it is not clear to what extent the measures in this guide are implemented in Ecuador or if there are resources at the local level to implement them.

Moreover, health systems require evidence to guide the best health policies and programs, as well as mechanisms to collaborate with communities. This collaboration allows a more effective response to health needs and facilitates communities in mobilization of resources and active participation in promoting and managing their health [3]. Authors have pointed out that health interventions also require implementation assessments to understand all the processes involved in the adoption and implementation of public health interventions and contextual factors which determine the outcomes of global health interventions [14].

This protocol, together with a previously published one [15], is part of a 5 year (2019–2023) European Research Council (ERC)-funded research project, “Contextualizing Evidence for Action in Diabetes in low-resource settings” (CEAD). It is an implementation science project whose overall objective is to explore the process by which global recommendations can be translated into context-specific, evidence-informed action for diabetes prevention in low-resource settings.

## 2. Materials and Methods

### 2.1. Research Goals

This study aims to evaluate the implementation of comprehensive diabetes care in two low-resource settings with different cultural, territorial, economic, and social conditions in Ecuador and to stimulate context-led health systems innovations to improve diabetes care and reduce inequity.

Specific aims:

1. To evaluate the implementation of diabetes care according to the 2017 Clinical Practice Guideline [12] in two health districts in Ecuador.
2. To use local knowledge and opinions to understand the observed weaknesses, their impact on health equity and identify how local health systems might be strengthened.
3. To assess the feasibility and effectiveness (in the short-medium term) of context-led health systems innovations to improve diabetes care locally.

### 2.2. Setting

We will carry out the fieldwork for both quantitative and qualitative research in two low-resource settings in Ecuador with different territorial, cultural, social, and economic characteristics. We selected 2 health districts in the country: (1) District 17D06, Quito, an urban health district with 507,499 residents (est. 2017); and (2) District 08D02, in Esmeraldas Province, a rural area in the northern coastal region of the country with a population of 44,498 (est. 2017). The choice was based on several characteristics. Firstly, the capital and coastal areas of Ecuador have a high diabetes prevalence according to national statistics [16]. Secondly, the two regions chosen have vastly different socio-economic, environmental, and cultural profiles. Lastly, to improve the feasibility of the research, we focused the research in areas where we had contact with local researchers who were motivated to implement evidence-based diabetes care in the population.

### 2.3. Study Design

**Figure 1:**
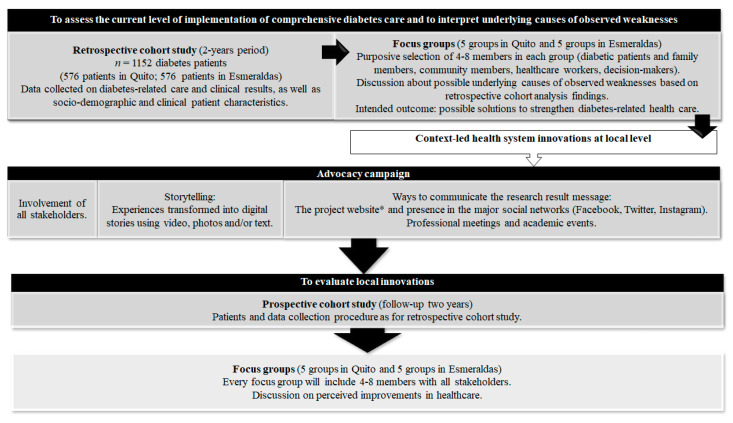
Study design and description of the phases that constitute it. * The project website is https://www.ceadproject.eu/ accessed on 15 January 2021.

This is a mixed-methods study (registered 16 September 2019, NCT04560062). Firstly, we will carry out a retrospective cohort study to assess the current level of implementation of comprehensive diabetes care over a 2 year period (from January 2019 to December 2020), by describing the healthcare received (process) and the health outcomes of a representative sample of diabetes patients currently accessing healthcare in the study regions. Secondly, we will interpret underlying causes of the observed weaknesses through focus groups prompted by the findings of the cohort study. Finally, the findings from the retrospective cohort study and qualitative research will generate local innovations which will be evaluated through a prospective follow-up of the same cohort with the participants included in retrospective cohort. The innovations in the health system will not be funded by the research and operational aspects will not be supported by the study team (Figure 1).

#### 2.3.1. Retrospective Cohort Study

- Study population:

Eligibility criteria:

The evaluation of comprehensive diabetes care will be undertaken on individuals diagnosed with type 2 diabetes aged over 18 years, who are currently accessing diabetes care in health facilities (defined as at least 1 visit of diabetes control in the 12 months prior to 1 January 2019) in the two study districts. Pregnant women will only be excluded if they are diagnosed with gestational diabetes or diabetes type 1.

Sample size and recruitment procedure:

We will enroll 1152 diabetes patients (576 per setting). The sample size is proposed conservatively to ensure we have precision to estimate outcomes of 50% with an absolute precision of ±5%, assuming a design effect of 1.2 and potential loss of 20%. A representative sample for each district will be obtained by stratified single-stage cluster sampling. The clusters are health facilities for which a sample of patients will be randomly selected. The sample will be stratified by facility type. Organization of public health services in Ecuador includes 4 types of facilities: Ministry of Public Health (MSP, from its Spanish acronym) facilities, social security facilities for affiliated workers, facilities dedicated exclusively to police forces and, finally, military hospitals/clinics. The latter three will be grouped together in a single stratum labelled a “complementary public health network”. Patient sampling will use the electronic consultation registry of each selected facility where possible. In rural areas, we will use an internal diabetes database of the Centre of Community Epidemiology and Tropical Medicine (CECOMET, from its Spanish acronym). We will first establish a list of patients diagnosed with type 2 diabetes currently accessing care, then draw a random sample of diabetes patients proportional to the total number of current patients at each facility within each facility type.

- Data collection procedure:

Research assistants will obtain data mainly from health services records, which will be supplemented by patient interviews. Community health workers working with the CECOMET and MSP will be tasked to collect data in the District 08D02, in Esmeraldas Province. In District 17D06 in Quito, data will be collected by trained interviewers that will contact the patients identified at the health service sample. Firstly, after obtaining informed consent they will carry out the patient interview. It will include information on the patients’ socioeconomic and demographic situation (self-reported), their access to health services as well as some clinical aspects (both obtained from health service records) (Table 1). In addition, we will carry out two brief questionnaires: one regarding perceived social support (the Multidimensional Scale of Perceived Social Support [17,18] MSPSS) and another on health-related quality of life (the Diabetes Health Profile-18 [19]; DHP-18). We will also add to the interview questions about the healthcare provided to the patient (e.g., frequency of disease monitoring, glucose tests, and other medical examinations). We will perform a cultural and linguistical adaptation of the DHP-18 questionnaire and validate it prior to use.

Afterwards, we will collect data from electronic health service records. We will collect data regarding whether the patient attended health visits and underwent screening for diabetes complications as recommended in the 2017 CPG in Ecuador [12] and record the date and details of any complications experienced throughout the evaluation and other clinical data such as comorbidities, treatments, or patient management by a multidisciplinary healthcare team.

Patient files, laboratory, and pharmacy records will provide information on diabetes care, treatment, glycemic control and presence of comorbidities and diabetes complications. Data pertaining to the 2-year period from 1 January 2019 to 31 December 2020 will be extracted.

- Outcomes

The components of comprehensive diabetes care to be assessed were selected from the 2017 CPG in Ecuador [12]. The section on pre-diabetes is not included. We will not assess the appropriateness of pharmacological treatments. The health professionals included in the assessment will be multidisciplinary at the second and third level of care.

Primary outcomes (Table 2) are a collection of basic indicators and are divided into two sections:

1. Disease control: Defined in two dimensions. Firstly, biochemical control based on the last laboratory result of glycated hemoglobin or blood glucose, recorded in the year of study. The second dimension will be health-related quality of life, assessed using the DHP-18 questionnaire previously validated in Ecuador.
2. Access to healthcare: Care process indicators that represent the proportion of the patients who undergo the care processes recommended by the CPG to manage the disease. We will obtain the care frequency for each patient as a quantitative value and will also categorize those care indicators that are recommended with greater periodicity in the CPG.

Secondary outcomes include:

3. Control of complication risk factors: Care result indicators that determine proportion of patients who meet the standards of diabetes care and the clinical objectives during the year of study (January 2019 to December 2020).
4. Measurements of diabetes-related behaviors/conditions: Access to medication (self-reported) and social support obtained from a standardized questionnaire, and patient information received, self-reported.
5. Resources used due to decompensation of the disease and/or complications.
6. Disease complications (as indicators that measure the proportion of patients with complications occurring that could result from insufficient control of the disease).

More detailed information on outcomes is available on the ClinicalTrials (NCT04560062).

- Analysis

In the quantitative analysis of comprehensive diabetes care, we will include descriptive statistics according to variable type and will calculate the proportion of patients that received care as per the CPG recommendations and/or the proportion receiving an intermediate level of care (as required). Proportions will be described with 95% confidence intervals. Variation in healthcare received and diabetes-related health will be described using sociodemographic and clinical characteristics of the patients to highlight potential inequities. A multivariate logistic regression model may be used to explore the relationship between the primary outcomes and socioeconomic explanatory variable and/or type of health facility. If necessary, we will adjust for potential confounders such as patient factors (e.g., sex, age, comorbidity, perceived social support) and/or environmental factors (e.g., proximity to the health center, availability of different medical specialties or methods as laboratory test).

Steps will be taken to prevent missing data (e.g., we will access data from different sources such as records of primary, specialized and hospital care), but some level is unavoidable and we will incorporate methods analyzing missing data or data from uncertain sources when necessary [20,21].

Statistical analysis will be performed using Stata Version 15 (StataCorp LP; College Station, TX, USA).

#### 2.3.2. Focus Groups

There will be two sets of 10 focus groups (FGs) each, 5 in urban (Quito) and 5 in rural (Esmeraldas) settings. The findings of the previous retrospective cohort analysis will be discussed in the first set of focus groups to interpret underlying causes of the observed weaknesses and will promote possible solutions to strengthen diabetes-related healthcare. The application of these possible solutions will be evaluated with data about healthcare received and clinical outcomes from electronic records and other related factors such as quality of life obtained in a prospective cohort study followed up after three years.

**Figure 2:**
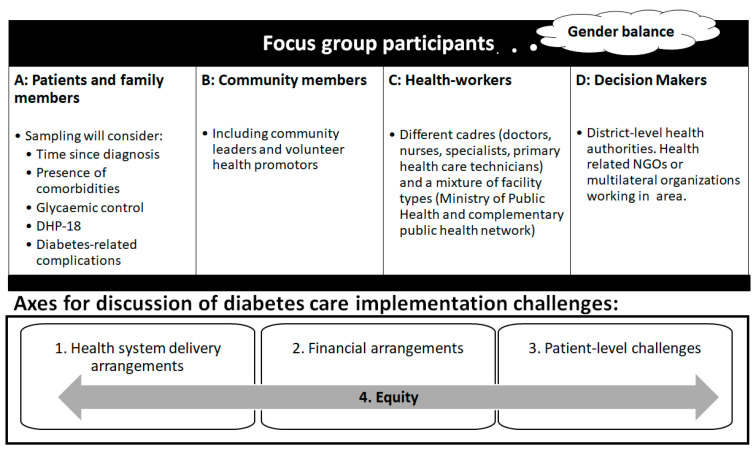
Purposive selection criteria for focus group participants and axes for discussion of diabetes care implementation challenges.

The second set of FGs will be prompted by the findings from the prospective cohort. Participants will discuss perceived improvements in healthcare using the same framework (Figure 2). The discussion will consider the success/failure of any context-led innovations occurring throughout the research, including experience and difficulties of formulating and implementing innovations. Selection of participants and procedure will be the same for both sets of FGs.

- Participants

Every focus group will include 4–8 members. They must be over 18, residents in the study area, and they must provide written informed consent. Participants will include community members (type B), healthcare workers (type C), decision-makers (type D) and diabetic patients and family members (type A). Patients will be selected among the quantitative study participants (cohort studies). Four FGs will include participant types A and B only; 2 groups with A, B and C, 2 groups with B, C and D, and 2 FGs with careful selection of all participant types. (Figure 2).

- Procedures

Prompts for FGs will include the findings of the quantitative healthcare evaluation described above. Health systems weaknesses identified will be summarized in a simplified evidence brief. The facilitator will guide discussion to enquire as to why these weaknesses may exist, what challenges exist for the stakeholders and how evidence-based recommendations may be better implemented, including a trade-off between a less stringent level of requirement in the application of evidence and better coverage and the whole effectiveness of the diabetic care program. In terms of the latter, the facilitator will guide discussion using a framework loosely based on implementation/adaptation/contextualization initiatives from the international knowledge base [27,28,29]. Participants will share their opinion and experiences in 4 broad axes (Figure 2). Participants will be prompted to suggest potential solutions. For example, among health service delivery arrangements, they may identify potential cadres for additional training and task sharing. In the FGs involving patients and family members, we will discuss perceived and/or experienced problems of access to/or utilization of services.

- Analysis

At least two members of the research team will be present at the FG discussions; one will have an observatory role, quietly noting observations related to body language of the speakers, time points and his/her perception of the power dynamic. All FG discussions will be audio-recorded and transcribed. Digitized transcripts will be analyzed using thematic analysis, assisted by Atlas.ti 8.0 (Scientific Software Development GmbH, Berlin, Germany).

#### 2.3.3. Transforming Findings into Innovations—Advocacy Campaign

Findings from the retrospective cohort and FGs will be transformed into context-led innovations using a co-creation process. This will involve the constructive exchange of different kinds of knowledge, resources, competences and ideas to transform the understanding of the problems and develop new ways of solving them. Implementation of social innovation requires a mutual process where all partners are equally involved in the co-creation of innovation. The participation of local decision makers, community and health professionals in this process will guarantee that proposed innovations are implemented at local level [30,31].

The steps that we have considered to make changes happen [32] are the following:

1. Getting the facts (research and data collection): The study will generate context-specific knowledge on the implementation of diabetes recommendations in participating communities.
2. Building support: The participation of stakeholders (patients and family members, community members, healthcare workers and decision-makers) in discussion groups will enable us to influence public policy as well as health services and community attitudes. This will motivate the community-based movements to improve the situation and to implement context-specific policies that prevent morbidity and mortality from diabetes and avoid new cases.
3. Developing goals and strategies: The objectives and strategies will emerge from the discussion groups with the participation of stakeholders. Therefore, this strategy will have a higher likelihood of success than externally imposed ones. As we have previously described, we will evaluate the possible improvements through changes in the pre-defined outcomes (Table 2).
4. Communicating our message: The project has several ways to reach the largest possible audience (both in the community and in decision-makers) as described below.

Communication will be developed through an advocacy campaign which includes the following proposals:

- Storytelling:

The storytelling component involves selection of champion storytellers from within the pool of participants of the FGs, as has been done in other studies [33]. Their experiences will be transformed into digital stories using video, photos and/or text. The participants may be selected from any of the FG discussions and the activity is relevant for the overarching goals of the project.

- Ways to communicate the research results:

Our objective is to reach all stakeholders (patients, health workers, communities, and decision-makers). We will encourage interaction with stakeholders through sharing and discussing findings at professional meetings (e.g., county health meetings) and academic events at the participating universities. This will be a two-way process that will encourage the generation of contextually tailored evidence for the Ministry of Public Health in Ecuador, as well as generalized tools that can help contextualize local policy options in other low-resource settings.

The most powerful tool for reaching a large audience, the internet, will allow us to reach the community as well as institutions. We have a website (https://ceadproject.eu/ accessed on 15 January 2021) that provides ongoing information regarding our research. It also gives information about our campaign goals, plans, and identity as well as about how to get involved, contact, and contribute to the campaign. We will use social media to encourage circulation, interpretation, and contestation of knowledge in both professional and lay networks in order to encourage changes in the community.

Furthermore, we will organize activities to transfer, share, and disseminate research results. For example, one activity with two phases, a scientific meeting with CEAD project collaborators and an event coinciding with World Diabetes Day (14 November), will include activities aimed at the general public. Activities will disseminate research results to date and provide an alternative and complementary view on diabetes and the difficulties of its daily management in low-resource settings, as well as solutions that can be provided. We understand that culture is the most suitable tool for spreading science and promoting the participation of citizens in knowledge creation and interpretation. Therefore, we have planned to carry out theatre activities, narrative exhibitions, music, and installation of panels which explain the project and the results obtained to date, as well as the next research objectives, in an urban area. The activities will also be recorded on video to facilitate their better diffusion.

#### 2.3.4. Cohort Follow-Up

- Study population:

The patients selected for the retrospective cohort study (described above) will form the prospective cohort population.

- Data collection procedure:

Data collection tools will be identical to those used for the retrospective cohort study. Clinical files will be reviewed every 12 months for the duration of follow-up (2 years) (Table 1).

- Outcomes:

Outcomes will be the same as for the retrospective cohort study (Table 2). We will assess changes in the indicators selected during the follow-up study.

- Analysis:

Any innovation in packets of care offered and how to organize healthcare services will be recorded and described in detail. Analysis will center on changes in the core indicators over time. Because this is not a controlled randomized intervention, we will not attempt to test whether changes are statistically significant.

### 2.4. Ethics and Dissemination

The study protocol has been reviewed and approved by the Universidad Miguel Hernández (UMH) project evaluation board (registration number 2018.291.E.OEP) and the nationally accredited ethical board at the Universidad Central del Ecuador (UCE, reference 00022-UMHE-E-2019), and ethical clearance has been provided by the European Research Council Executive Agency (ERCEA, Ref. Ares (2018)5827042-14/11/2018). An independent Ethics Adviser has been appointed and we will monitor ethical and participatory issues, paying particular attention to gender and other equity concerns throughout the research.

Furthermore, written authorization to conduct the study and review clinical records in public health facilities has been obtained from the National Health Intelligence Directorate of the Ministry before any data collection in Ecuador will take place.

At the beginning of the study, we will establish two project advisory committees composed of stakeholders. They will represent the participants’ interests and address the risk of vulnerability/stigmatization. A priori, considering the research nature and the disease involved, we do not believe that there is a significant risk of stigmatization. However, the committee and research team will meet regularly throughout the study period to obtain information about patient and community concerns and to monitor ethical and participatory problems.

Regarding knowledge dissemination, results will also be presented in scientific conferences and seminars and will be written up for publication in peer-reviewed journals. In addition, we have designed a dissemination plan. We provide more detail on this plan above (see Section 2.3.3 “Transforming Findings Into Innovations—Advocacy Campaign”).

The CEAD will archive datasets generated by the project in the data repository Zenodo and will be linked in the study website (https://ceadproject.eu/ accessed on 15 January 2021). Zenodo will assign a Digital Object Identifier (DOI) and we will include DOI of (meta) data in the relevant publications to find the raw (but de-identified) data easily. Raw data will be freely available after publication of research articles, including the transcripts of the interviews and focus groups.

## 3. Discussion

This study will provide insight into the factors currently responsible for poor control rates of NCDs in resource-limited settings and will capture the attitudes and opinions of both patients and physicians on the care and treatment of chronic diseases. The mixed-method approach seeks to reveal how things that appear possible in theory can be transformed into reality in practice [34,35]. We hope to demonstrate a process of eliciting change by generating locally relevant knowledge specific to the problem at hand and involving local stakeholders in the development of improvements that are relevant and applicable in their context.

Substantial gaps in the global landscape of health research in LMICs [6], and the alarming predictions associated with NCDs, makes providing knowledge about how to implement diabetes recommendations in these settings an urgent priority.

The outbreak of the coronavirus COVID-19 pandemic has led to the adaptation of research methodologies to a global health emergency context (e.g., in some cases we may conduct the interviews by telephone, and the focus groups will be postponed and conducted with appropriate social distancing measures) and has inevitably influenced the context we seek to study. This is the most important global health crisis of our time. However, it also has the potential to create devastating social, economic, and political crises [36]. Ecuador is the country with the second-highest incidence of coronavirus cases in South America [37]. The pandemic could accentuate some public health problems, because it may increase demand for resources which could lead to a collateral rise in mortality from other causes [37]. The increase in indirect crisis-related deaths has already been observed in other resource-poor countries struck by an epidemic [38]. Moreover, a post-COVID-19 global economic slump, which could lead to a further deterioration in health equity [39,40]. Therefore, adequate public policy responses are essential this time to ensure that the COVID-19 pandemic does not increase health inequalities for future generations [40]. We consider that the results of our research will also provide knowledge regarding the capacity of the health system to be resilient, as well as providing possible solutions for the management of diabetes patients in the context of scarce resources.

The strengths of this study are the inclusion of both rural and urban areas to capture the experiences of patients with diabetes, and a sample size that will allow robust evaluation of results. The methodology, which combines quantitative and qualitative methods, will allow more complete results to be obtained. However, this study is not without limitations. First, we will retrieve some data from medical files, so the quality of the data collected is highly dependent on the quality of the information recorded in the files. Thankfully, we will be able to confirm any important missing data in the medical history through the patient interviews.

## 4. Conclusions

Our study hopes to generate better possible solutions to the weaknesses of diabetes-related healthcare in low resource settings. In addition, it will represent an important source of data that will be public and accessible to help understand how we can empower communities to develop innovations in their care protocols that are tailored to their context and applicable to the realities in which they live. Finally, although we are confronted with a scenario not known in recent history, we think that our research is even more necessary given the impact of the pandemic on resource-poor health systems, hopefully contributing to the reduction of indirect morbidity and mortality from the crisis.

## Figures and Tables

**Table 1:** Summary of routinely collected data.

Type of Data		Time Point			
Category	Specific Information	Baseline	Retrospective (2 years)	Prospective	
				Year 1	Year 2
Demographic data	Date and country of birth	X			
	Sex	X			
	Ethnicity	X			
	Marital status	X			X
Socioeconomic data	Education	X			X
	Employment status	X			X
	Household income	X			X
Data on access to health services	Primary care consultations		X	X	X
	Specialist consultations ^1^		X	X	X
	Number of biochemical analysis ^2^		X	X	X
	Number of blood pressure records		X	X	X
	Number of BMI records		X	X	X
Clinical data	Date of diabetes diagnosis	X			
	Diabetes-related hospitalizations		X	X	X
	Diabetes-related medical assistance		X	X	X
	Biochemical results ^2^		X	X	X
	Results of physical measurement		X	X	X
	Medications ^3^		X	X	X
	Diabetes complications ^4^		X	X	X
Access to treatment (self-reported) ^5^		X			X
Perceived social support (MSPSS) ^5^		X			X
Health-related quality of life (DHP-18) ^5^		X			X

^1^ Nutritionist, psychologist, and/or physiotherapy. ^2^ Glucose, glycated hemoglobin, lipid profile (cholesterol, triglycerides, HDL, LDL), renal function (creatinine, glomerular filtrate, albuminuria). ^3^ Treatment of diabetes or other chronic pathologies. ^4^ Retinopathy and/or blindness, lower limb amputations, cardiovascular events, and renal dysfunction. ^5^ Reassessment in the final 6 months of follow-up. Abbreviations: BMI: body mass index MSPSS: The Multidimensional Scale of Perceived Social Support, DHP-18: The Diabetes Health Profile-18.

**Table 2:** Study outcome measures.

Primary Outcomes		Secondary Outcomes	
Outcome	Definition	Outcome	Definition
**A. Disease control**		**C. Control of complication risk factors over the 12-month period**	
1. Biochemist	Proportions of patients with biochemically controlled diabetes ^3^	1. Blood pressure control	Proportion of patients with blood pressure < 140/90 [12]
2. Health-related quality of life	Individual score of the Diabetes Health Profile-18 [19].	2. Weight control	Proportion of patients with BMI between 18.5 kg/m^2^ and 25 kg/m^2^ or 5% weight loss [12]
**B. Access to health care of diabetes recommended by the CPG** [12] **over the 12-month period**		3. Lipemic control	Proportion of patients with LDL cholesterol level < 100 mg/dL [12]
1. Consultations with a GP ^1^	Indicators will expressed as number of measurements per patient and will also be categorized according to compliance with CPG recommendations.	4. Renal health	Proportion of patients with microalbuminuria level <30 mg/day [12,22]
2. Glycemic testing ^1^		**D. Diabetes-related behaviours/conditions**	
3. Blood pressure records ^1^		1. Perceived social support	Individual score from the Multidimensional Scale of Perceived Social Support.
4. BMI determination ^1^		2. Access to treatment	Information self-reporting.
5. Waist circumference record ^1^		**E. Resources used due to decompensation of the disease and/or complications over the 12-month period**	
6. HbA1C measurement	Indicators will be expressed as proportion of patients with minimum measurements considered acceptable.	1. Unscheduled consultations ^1^	Number of unscheduled medical appointments required due to diabetes or a diabetes complication.
7. Lipid profile determination			
8. Creatinine determination		2. Hospitalizations	Indicator will be reported as proportion of persons requiring hospitalization owing to glycemic decompensation and as total number of hospitalizations.
9. Diabetic foot exam			
10. Odontological assessment		**F. Complications**	
11. Fundus examination ^2^		1. Renal dysfunction ^4^	Proportion of patients with renal dysfunction. According to last glomerular filtration rate and serum creatinine values in pregnant women [23].
12. Microalbuminuria determination			
13. Erectile dysfunction assessment			
14. Cardiovascular screening		2. Eye disease	Proportion of patients with retinopathy and/or blindness.
15. Diabetes-related education	Proportion of patients who have received diabetes education (information self-reporting)	3. Amputations	Proportion of patients with lower limb amputations.
		4. Cardiovascular disease	Proportion of patients with 1 or more cardiovascular events ^2^.

^1^ We will provide both the continuous and categorized value for data analysis. ^2^ Evaluation period: time since diabetes diagnosis. ^3^ Controlled diabetes: HbA1C < 7% or HbA1C < 8% if ≥15 years of evolution or complications and serious comorbidities; or fasting blood glucose: 70–130 mg/dL or postprandial blood glucose < 180 mg/dL [12,24]. Pregnant women: fasting blood glucose: 60–99 mg/dL or postprandial blood glucose: ≤140 mg/dL (1 h after eating) or ≤120 (2 h after eating) [25]. ^4^ MDRD-4 formula not validated for pregnant women [26]. Abbreviations: GP: general practitioner; CPG: Clinical Practice Guideline; HbA1C: glycated hemoglobin; BMI: body mass index; HDL: high-density lipoprotein; LDL: low-density lipoprotein.

## Data Availability

No new data were created or analyzed in this study. Data sharing is not applicable to this article.
